# Thermostable Hexameric Form of Eis (Rv2416c) Protein of *M. tuberculosis* Plays an Important Role for Enhanced Intracellular Survival within Macrophages

**DOI:** 10.1371/journal.pone.0027590

**Published:** 2011-11-16

**Authors:** Arsheed A. Ganaie, Ravi K. Lella, Rahul Solanki, Charu Sharma

**Affiliations:** 1 CSIR-Institute of Microbial Technology, Chandigarh, India; 2 Drug Target Discovery and Development Division, CSIR-Central Drug Research Institute, Lucknow, Uttar Pradesh, India; National Institute of Allergy and Infectious Disease, United States of America

## Abstract

Eis protein is reported to enhance the intracellular survival of *Mycobacterium tuberculosis* in human macrophages. Eis protein is not only known to skew away the immunity by disturbing the protective T_H_1 response, but aminoglycoside acetyltransferase activity of Eis is reported to regulate autophagy, inflammation and cell death. Here we have gained insight into the structure-function properties of Eis. Eis protein is a hexameric αβ protein. Although urea and guanidinium hydrochloride (GdmCl) was found to induce one-step unfolding of Eis but size exclusion chromatography showed that GdmCl treated Eis maintained its hexameric form. SDS-PAGE assay confirmed that hexameric form of Eis is partially stable to SDS and converts into trimers and monomers. Out of these three forms, aminoglycoside acetyltransferase activity is found to be associated only with hexamers. The Tm of Eis was found to be ∼75°C. Aminoglycoside acetyltransferase Eis demonstrated remarkable heat stability retaining >80% of their activity at 70°C which falls down to ∼50% at 75°C and is completely inactive at 80°C. Further, intracellular survival assay with heated samples of *M. smegmatis* harboring *eis* gene of *M. tuberculosis* H37Rv demonstrated a possible role for the thermostability associated with Eis protein in the enhanced intracellular survival within macrophages. In sum, these data reveal that only hexameric form of Eis has a thermostable aminoglycoside acetyltransferase activity. This is the first report showing the thermostability associated with aminoglycoside acetyltransferase activity of Eis protein being one of the essential features for the execution of its biological role.

## Introduction

*Mycobacterium tuberculosis* can successfully survive inside the host macrophages in spite of the antimicrobial effector functions of the macrophages [1]–[4]. A variety of mechanisms have been suggested to contribute to the survival of *M. tuberculosis* within macrophages [5], [6].

Eis protein (Rv2416c of *M. tuberculosis*) is a secretory protein of 42.0 kDa which provides enhanced intracellular survival to *M. tuberculosis* in the human macrophage cell line U-937 [7]. Eis protein has the aminoglycoside acetyltransferase activity and belongs to GCN5-related family of N-acetyltransferases (GNAT) [8], [9]. Members of the GNAT family of proteins are involved in a variety of activities, ranging from transcriptional activation to antibiotic resistance [10]. The over-expression of Eis aminoglycoside acetyltransferase due to the mutations in the -10 and -35 promoter region was found to confer kanamycin resistance [8]. Kanamycin is one of the important second-line anti-TB drugs which inhibit protein synthesis [11].

Eis is reported to modulate the secretion of TNF-α and IL-10 by primary human monocytes [12] and skews away the immunity by disturbing the protective T_H_1 response [13]. Shin et al (2010) reported the function of Eis as a regulator of autophagy, inflammation and cell death which ultimately suppresses host innate immune defenses [14]. Although the information about the mechanism(s) by which Eis executes its function has started pouring in, the details of the function and the structural characterization of Eis protein are not known.

To address this issue, the current study examined the structural and functional properties of Eis. Eis exists as hexamers with 28% α helix, 19% β sheet and 53 % random coils at room temperature (25°C). Although the unfolding induced by urea and guanidinium hydrochloride (GdmCl) was found to be one-step process but size exclusion chromatography (SEC) showed that GdmCl treated Eis maintains its hexameric form. The Tm of Eis was found to be ∼75°C. Further, SDS assay studies suggest that SDS significantly converts the hexamers into trimers and monomers. Only hexameric form of Eis has aminoglycoside acetyltransferase activity with remarkable thermostability. The thermostability associated with Eis protein is responsible for enhanced intracellular survival at the higher temperature. To summarize, this is the first report characterizing the role of thermostability associated with of aminoglycoside acetyltransferase Eis protein of *M. tuberculosis*.

## Results

### Eis protein is hexameric in nature

**Figure 1:**
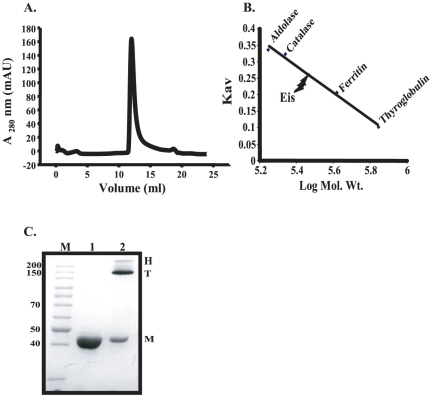
Size exclusion chromatography for determination of molecular weight and oligomeric stability. Panel A. Size-exclusion chromatographic profile for Eis on Superdex 200HR 10/30 column in 20 mM phosphate buffer, pH 8.0 at 25°C. Panel B. standard molecular weight markers Aldolase (158 kDa), Catalase (219 kDa), Ferritin (416 kDa), Thyroglobulin (699 kDa) run under the same conditions for molecular weight determination. Panel C. SDS-PAGE resistance assay. lane 1: unboiled and lane 2: boiled Eis protein. Equal amount of protein was loaded in lanes 1 and 2. H: Hexamers; T: Trimers and M: Monomers. The experimental details are given in materials and methods.

We amplified the *eis* gene from the *M. tuberculosis* H37Rv genomic DNA and cloned, over expressed and purified using Ni-NTA chromatography as described previously [13]. The purified protein migrated as a single band corresponding to ∼46 kDa on SDS-PAGE. Gel filtration of the recombinant Eis protein on a calibrated Superdex™ 200HR 10/30 column eluted as a single discrete peak at a retention volume of 12.0 ml (Fig. 1A). When the elution volumes of the marker proteins were plotted as a function of log of molecular weight (Fig. 1B), the Eis protein was found to have a molecular mass of about ∼280 kDa. During gel filtration no other peaks were observed, further verifying the homogeneity of the purified protein. The results of the subunit mass, as determined by SDS-PAGE, along with size exclusion chromatography (SEC) studies demonstrate that Eis protein of *M. tuberculosis* exists as a homo-hexamer under experimental conditions.

### Eis hexamers are partially stable to SDS

To further explore the stability of oligomeric form, boiled and unboiled protein samples of Eis containing SDS were electrophoresed on SDS-PAGE (Fig. 1C). In case of unboiled samples three forms of Eis protein, namely, monomers (M), trimers (T) and hexameric (H) forms were observed while under boiled conditions, only monomers were detected. The presence of trimeric form indicated that hexameric form might be partially stable in the presence of SDS and is converting into trimeric forms. Further, it was observed that trimers are the most predominant form than the other two.

### Structural features of Eis Protein

**Figure 2:**
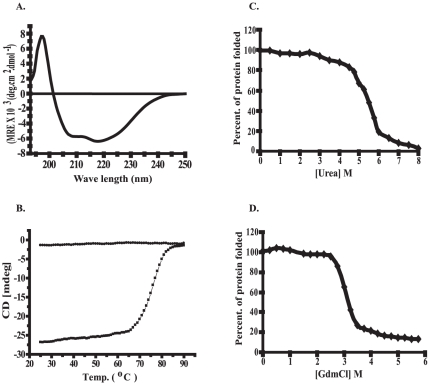
Secondary structural changes of Eis in the presence of different denaturants analyzed by CD. Panel A. Far-UV CD spectra of native recombinant Eis protein. Panel B. Temperature induced changes in secondary structure of Eis as monitored by following changes in molar ellipticity at 222 nm obtained from far-UV CD curves at increasing (▪) and decreasing (•) temperature. Changes in secondary structure of Eis due to Urea and GdmCl as monitored by following the changes in ellipticity at 222 nm obtained from far-UV CD curves at increasing concentrations of Urea (Panel C) or GdmCl (Panel D).

Studies on the model polypeptides and proteins revealed that the α-helical and β-sheet proteins show characteristic far-UV CD spectra. The α-helical proteins have two minima at 222 nm and 208 nm and the β-sheet proteins have a single minimum at 216 nm [15]. For Eis protein, far-UV CD spectrum characteristic of a protein having both α-helical and β-sheet secondary structure was observed at 25°C (Fig. 2A). The program K2D (www.embl-heidelberg.de/~andrade/k2d.html) was used to analyze the CD data, in order to calculate the amounts of secondary structural elements comprising this domain. The percentage of α-helix, β-sheet and random coil is calculated to be 28%, 19% and 53% respectively. These results give a primary indication of the structure of the expressed protein.

The thermal unfolding of Eis was characterized by monitoring the loss of secondary structure at increasing temperature. Fig. 2B summarizes the changes in CD ellipticity at 222 nm for Eis protein at increasing temperature from 25°C to 90°C. The Eis protein undergoes a co-operative thermal transition. It was found to be stable up to 60°C and completely denatured at ∼80°C. The T_m_ (denaturant temperature where 50% denaturation of protein is observed) of the Eis protein was ∼75°C. Furthermore, the transition was found to be irreversible on cooling of the Eis protein from 90°C to 25°C after the heating scan.

### Changes in molecular properties of Eis associated with Urea and GdmCl-induced unfolding

The urea and GdmCl induced changes in the secondary structure of Eis were studied by monitoring changes in CD ellipticity at 222 nm at increasing concentrations of urea and GdmCl. A sigmoidal dependence of decrease in ellipticity at 222 nm with increasing concentrations of urea was observed. No change was observed for the native enzyme up to ∼3.0 M Urea (Fig. 2C). However, between 3.0 and 6.5 M Urea, there was a decrease in ellipticity value at 222 nm and a complete loss of signal above 8.0 M urea was observed. These observations suggest that higher concentrations of Urea (≥7.0 M) induce complete unfolding of Eis protein. A Cm (denaturant concentration where 50% denaturation of protein is observed) of ∼5.25 M Urea was found to be associated with this transition.

With increasing concentrations of GdmCl, a sigmoidal dependence of decrease in ellipticity at 222 nm was obtained, as is the case with Urea. A single transition in the GdmCl concentration range between 2.5 M and 3.5 M was observed (Fig. 2D). Moreover, ∼80% of the protein was observed to lose its secondary structure at 4.0 M GdmCl and ∼20% of the protein was found to be present as folded even after the treatment with 6.0 M of GdmCl. A Cm of about 3.15 M GdmCl associated with this transition indicates the rigidity of the structure.

### Changes in quaternary structure of Eis during GdmCl-induced unfolding

**Figure 3:**
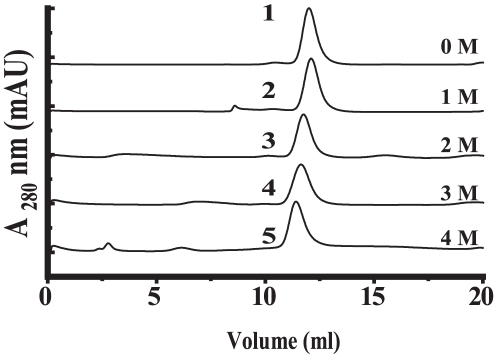
Size exclusion chromatographic profiles forGdmCl treated Eis on superdex 200HR 10/30 column. The curves 1, 2, 3, 4 and 5 represent profiles for Eis incubated in 0 M, 1 M, 2 M, 3 M and 4 M GdmCl, respectively.

To confirm the result obtained in Fig 2D and the effect of GdmCl–induced structural changes on the quaternary structure of Eis, the effects of increasing GdmCl on the molecular dimension of Eis were monitored by SEC on Superdex™ 200HR 10/30 column. Fig. 3 summarizes the results of gel permeation experiments of Eis protein either in the absence or in the presence of increasing concentration of GdmCl at 25°C. For native Eis, a single peak centered at 12.0 ml, indicating the hexameric nature of Eis protein. However, for 1.0 M, 2.0 M, 3.0 M and 4.0 M GdmCl-treated Eis, a single peak with a retention volume of 12.0 ml, 11.76 ml, 11.64 ml and 11.41 ml, respectively, was observed. The result obtained with SEC analysis of GdmCl treated Eis protein confirmed that Eis is able to retain its quaternary structure and is in agreement with result shown in Fig 2D. The decrease in the retention volume with increase in the GdmCl concentration is indicative of a protein conformation with a larger hydrodynamic radius, i.e., relatively an unfolded protein.

### SDS stabilizes Eis protein

**Figure 4:**
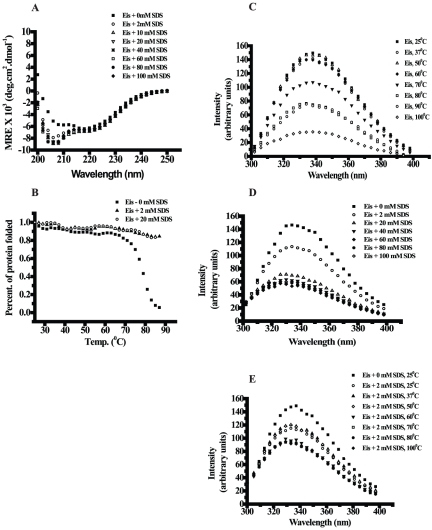
SDS induced changes in the Eis protein. Panel A. Far-UV CD curves of Eis measured in the presence of different concentrations of SDS ranging from 2 mM to 100 mM or in the absence of SDS. Panel B. Temperature induced changes in secondary structure of Eis either in the absence or presence of 2 mM and 20 mM SDS were monitored using CD by following changes in molar ellipticity at 222 nm. Panel C. The fluorescence spectra of Eis at different temperatures ranging from 37°C to 100°C. Panel D. The fluorescence spectra of Eis at different concentration of SDS. Panel E. The fluorescence spectra of Eis in the abscene or presence of 2 mM SDS at temperatures ranging from 25°C to 100°C.

The stability of Eis in the presence of SDS was characterized by CD and fluorescence measurements at increasing concentration of SDS (Fig. 4A–E). A CD spectrum of Eis in the presence of increasing concentration of SDS showed that Eis protein is highly stable in detergent environment. The far-UV CD spectra of Eis at increasing concentrations of SDS showed a significant decrease in ellipticity at 208 nm from 0 mM SDS to 20 mM SDS and remained unaltered up to 100 mM SDS. No change in the ellipticity at 222 nm was observed from 20 mM to 100 mM SDS (Fig. 4A). Normal thermal denaturation spectra of Eis show that T_m_ is approx. 75°C (Fig. 2B). But in the presence of 2.0 mM and 20 mM SDS, Eis protein is not showing any thermal denaturation (Fig. 4B), which further indicates that Eis is stabilizing towards thermal denaturation in presence of SDS.

### Intrinsic Tryptophan Fluorescence

The intrinsic tryptophan fluorescence spectra of Eis at increasing temperatures are shown in Fig. 4C. The fluorescence intensity at 339 nm decreased with increase in temperature up to 60°C followed by a gradual decrease from 70°C to 90°C (Fig. 4C). At 100°C, there is almost complete loss of fluorescence intensity. While maximum fluorescence intensity (λ_max_) at 339 nm was also found to be decreased from 2 mM to 20 mM, but increasing concentration of SDS from 20 mM to 100 mM did not exhibit any significant decrease (Fig. 4D). To see the combined effect of SDS and temperature, fluorescence spectra were recorded at increasing temperature and in the presence of fixed concentration of SDS (2 mM). In the presence of 2 mM SDS, a first sharp decrease in fluorescence intensity at 339 nm was observed with increase in temperature from 25°C to 50°C and then the second decrease was observed with increase in temperature from 60°C to 100°C (Fig. 4E). In the presence of 2 mM SDS, at 100°C, the complete loss of fluorescence intensity was abolished, which confirms that SDS stabilizes Eis towards the effect of heat.

### Only Hexameric form of Eis is responsible for thermostable Aminoglycoside Acetyltransferase activity

**Figure 5:**
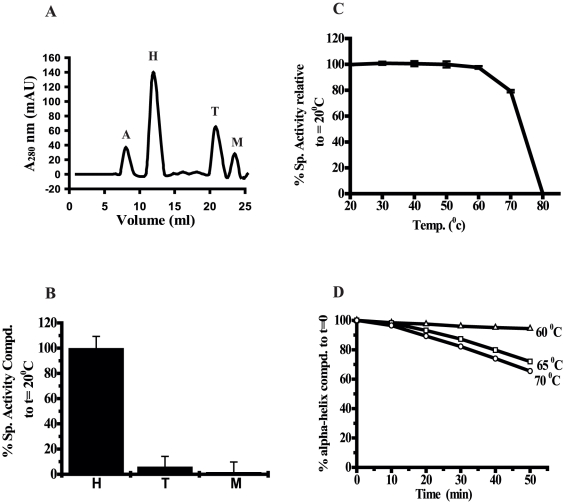
Thermostable aminoglycoside acetyltransferase activity of Eis. Panel A. Size exclusion chromatographic profile of SDS treated Eis. Peak A represents micelles of SDS; Peak H: hexamers; Peak T: trimers and Peak M: monomers of Eis. Panel B. Hexamers, trimers and monomers obtained from SEC in panel A were examined for aminoglycoside acetyltransferase activity. Panel C. Eis protein was incubated at increasing temperatures for 10 mintues and aminoglycoside acetyltransferase activity was assayed. The specific activity was determined over a linear range. The experiment for each sample was performed in triplicate and the mean ± S.D. is represented above the graph. Panel D. The effect of temperatures 60°C, 65°C and 70°C on the secondary structure of Eis protein was measured by recording the CD at the respective temperatures.

Eis is known to have acetyltransferase activity [8] and in Fig 1D we have shown that Eis exist as hexamers, trimers and monomers. We examined which of the three forms of Eis is responsible for aminoglycoside acetyltransferase activity. Fig 5A shows the SEC profile of SDS treated Eis protein. Eis protein was treated with 2.0 mM SDS for 3 hours at room temperature (24°C) and fractionated using calibrated Superdex™ 200HR 10/30 column. Aggregates of SDS micelles were obtained at 8.0 ml which is a void volume of the column. Discrete peaks of hexamers, trimers and monomers were eluted at 12.0 ml, 20.9 ml and 23.6 ml, respectively. Fractions having respective oligomers and monomers were collected and aminoglycoside acetyltransferase activity was tested. Only hexamers exhibited the aminoglycoside acetyltransferase activity. Trimers and monomers were found to be inactive (Fig 5B).

Eis is highly thermostable protein (Fig 2B and 4B) with Tm ∼75°C. Therefore, thermostability of Eis was assayed by studying both enzymatic activity and CD. The enzyme retained almost 80% activity at 70°C. The activity was reduced to 50% at ∼75°C and completely inactive at 80°C (Fig 5C). Further, upon incubation at 60°C, 65°C and 70°C (as determined by sample probe), the α-helices of Eis protein were very stable at 60°C and unfolded in a linear time-dependent manner at 65°C and 70°C (Fig 5D).

### Effect of temperature on the binding of Acetyl CoA to Eis

**Figure 6:**
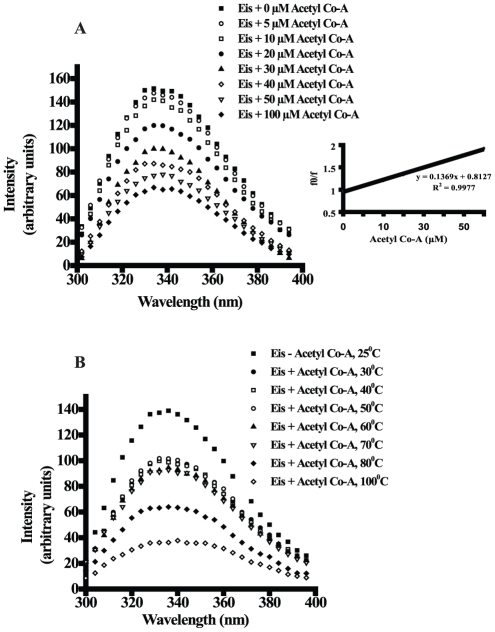
Effect of temperature on the Eis-Acetyl CoA interaction study. Panel A. Eis-Acetyl CoA interaction studies were done using increasing concentration of Acetyl CoA from 20 µM to 100 µM at 25°C and fluorescence emission spectra were monitored. Stern-Volmer plot used for calculation of binding constant (Ksv) for Acetyl CoA is shown on the right side in Panel A. Panel B. Eis-Acetyl CoA interaction studies were done by recording the fluorescence emission spectra with fixed concentration of Acetyl CoA (20 µM) or without Acetyl CoA but at different temperatures ranging from 25°C to 100°C as mentioned in materials and methods.

In order to check the binding of the Acetyl CoA to Eis at high temperatures, fluorescence quenching studies were performed as described in materials and methods. Fluorescence quenching studies demonstrated that the Eis binds to Acetyl CoA in a concentration dependent manner (Fig 6A). Quenching of intrinsic fluorescence was observed in presence of Acetyl CoA with Ksv constant of 0.02 µM^−1^. Notably, the quenching profile of intrinsic fluorescence at high temperatures ranging from 37°C to 70°C demonstrated that the binding of Acetyl CoA to Eis remained unaffected up to 70°C (Fig 6B).

### Survival of *M. smegmatis* harboring *eis* gene of *M. tuberculosis* at different temperatures

**Figure 7:**
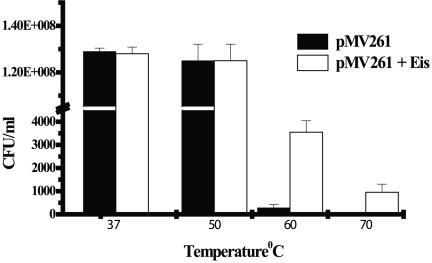
Survival profile of *M. smegmatis* harboring *eis* gene of *M. tuberculosis* H37Rv at different temperatures. Cells grown at 37°C to log phase were incubated at 37°C, 50°C, 60°C and 70°C for 10 minutes and survival was determined. Data are mean ± standard deviations from three independent experiments.

Viability of *M. smegmatis* harboring *eis* gene of *M. tuberculosis* H37Rv (*M. smegmatis*/Eis) grown at 37°C was determined after incubating at 37°C, 50°C, 60°C and 70°C for 10 minutes. *M. smegmatis* transformed with pMV261 vector was used as negative control. There was only a marginal loss of viability at 50°C. Vector transformed cells demonstrated drastic reduction in viability when cells were incubated at 60°C but *M. smegmatis*/Eis showed 11.4 fold enhanced survival. Although the survival due to Eis at 70°C is reduced drastically, the viability of vector transformed cells was not significant (Fig 7). Based on this result, intracellular survival due to *M. smegmatis*/Eis was assayed at 37°C, 50°C and 60°C.

### Intracellular Survival Assay

**Figure 8:**
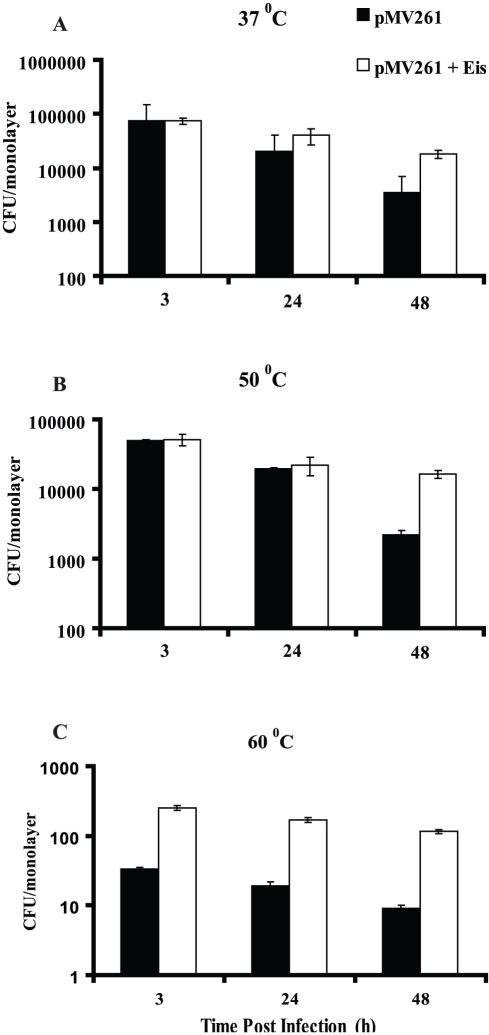
Intracellular survival of *M. smegmatis* harboring *eis* gene of *M. tuberculosis* H37Rv in THP1 cells. Survival of intracellular bacteria was counted at 3 h (Panel A), 24 h (Panel B) and 48 h (Panel C) post infection as per the details described in materials and methods. Data are mean ± standard deviations from three independent experiments.

*M. smegmatis* harboring either pMV261 vector or *eis* gene of *M. tuberculosis* heated to 37°C, 50°C and 60°C for 10 minutes was used for intracellular survival assay as described in methods. Eis mediated enhanced intracellular survival at 37°C was observed with 1.9 and 5.0 fold increase at 24 and 48 h post infection, respectively. This result is in complete agreement with the findings of Wei et al (8). With 50°C incubation, the survival benefit due to Eis increased to 7.4 fold at 48 h post infection. Although no benefit was seen due to Eis mediated intracellular survival at 3 hours at 37°C and 50°C but at 60°C, the Eis mediated intracellular survival at 3 h is 7.0 fold which increased significantly to 9.0 and 12.8 fold at 24 and 48 h, respectively (Fig 8).

## Discussion

Here we describe the previously uncharacterized structural features of the first aminoglycoside acetyltransferase Eis protein of *M. tuberculosis* systematically. This was investigated by using several systematic biophysical and biochemical methods to characterize the oligomeric state, thermostability and activity of Eis (SEC, CD, fluorescence, aminoglycoside acetyltransferase activity, binding of Acetyl CoA to Eis and intracellular survival assay). This is the first report that elaborates the importance of thermostability associated with Eis in the intracellular survival of Mycobacterium upon infection. The relevance of our results is discussed below.

Eis is a thermostable, hexameric α/β protein with Tm 75°C. GdmCl treated Eis was found to maintain its hexameric form with larger radii than native protein (∼11.4 ml vs 12.0 ml elution volume) which suggest that the hexameric forms have been opened up which is possible only if GdmCl is inducing the denaturation of the protein at the secondary structures but leaving the quaternary structures intact. 20% of the folded Eis protein obtained upon GdmCl-induced denaturation is sufficient to retain the hexameric form. Although hexamers were GdmCl resistant but they were found to be partially labile to SDS in SDS-PAGE assay. The Cm value of GdmCl-treated Eis protein is an indicative of structural rigidity.

According to the primary amino acid sequence, Eis protein has 7 tryptophan residues at positions 53, 76, 231, 237, 293, 328 and 335. The maximum emission wavelength of the tryptophan fluorescence for the recombinant Eis protein was observed at 339 nm. The buried and the exposed tryptophan residues in folded protein show fluorescence emission maxima at 330–340 nm and 348–356 nm, respectively [16]. The SDS and temperature induced alterations in the tertiary structure of Eis protein studied by monitoring the changes in the tryptophan fluorescence emission maxima of Eis protein show that the tryptophan molecules are likely to be buried as even the cumulative effect of SDS and temperature failed to induce the changes in the maximum emission wavelength. Taken together, the data obtained from the studies of effect of SDS on thermal denaturation and fluorescence studies reflect that SDS stabilizes Eis towards the effect of heat.

Eis is a thermostable protein which exists as hexamer-trimer-monomer and with the knowledge that Eis has an aminoglycoside acetyltransferase activity; we considered the possibility that either all the three forms or any one of them might be having aminoglycoside acetyltransferase activity. The current study confirms that only hexameric form of Eis has active aminoglycoside acetyltransferase activity. Eis has stable acetyl transferase activity with unaltered binding to Acetyl CoA up to ∼60°C. This is the first report showing that the aminoglycoside acetyltransferase activity of Eis protein of *M. tuberculosis* is highly thermostable. After this paper was submitted, a report directed at identifying the unprecedented ability to acetylate multiple aminoglycosides at many amines using structural and mutagenesis methods was published [17]. Chen et al [17] examined the X-ray crystal structure of acetyl transferase Eis protein and showed that Eis is a hexameric protein with a complex tripartite fold but has not explored the thermostability of Eis protein and its role in the intracellular survival of *M. tuberculosis* within macrophages.

The only reported acetyl and arylamine N-acetyltransferase (NAT) from *M. tuberculosis* belonging to the GNAT and NAT superfamilies are 2′-N-acetyltransferase (AAC-2′-Ic) [18], [19], [20] and TBNAT (NAT from *M. tuberculosis*) [21]. Although the crystal structure of 2′-N-acetyltransferase (AAC-2′-Ic) has been determined in the apoenzyme form and also in ternary complexes with drug [20] but its stability towards heat is not known. TBNAT is the only reported thermostable arylamine NAT with active oligomers of *M. tuberculosis* [21]. There is no other report available either on the biophysical, biochemical characterization or structural information of any other aminoglycoside acetyltransfearse of *M. tuberculosis* to date.

Thermostable proteins were predicted to be essential [21] for the intracellular survival as during the initial bacterial infection there is increase in internal temperature due to defense mechanism of the host against the pathogen [22]. Eis has targets in eukaryotic cells and regulates important functions of host upon infection [14] thus playing an important role in the enhanced intracellular survival in host macrophages [7]. We, therefore, examined the role of thermostability associated with aminoglycoside acetyltransferase activity of Eis protein in the intracellular survival within macrophages upon infection. *M. smegmatis* harboring *eis* gene of *M. tuberculosis* H37Rv is an excellent system for this study and has been used successfully earlier by Wei et al [7]. There is no report on the functional characterization of Eis protein in *M. smegmatis* to date.

While survival profile of *M. smegmatis* harboring *Eis* gene of *M. tuberculosis* at different temperatures very clearly demonstrated that Eis provides significant thermal protection at high temperatures up to 60°C but protection from heat was not observed in the pMV261 transformed *M. smegmatis* cells which was included as a control. The significant survival benefit due to thermal protection concurs with that of the intracellular survival within macrophages upon infection. The structural stability, acetyl transferase activity of Eis protein along with studies on intracellular survival within macrophage at high temperatures confirms that the thermostability associated with Eis protein has a very important role to play. Although the intracellular temperature is unlikely to soar beyond 45°C during initial bacterial infection, the thermostability associated with Eis protein not only prevents its inactivation but ensures its activity at this temperature with a safe margin. Given the important role played by thermostability of Eis protein in the intracellular survival of *M. tuberculosis* within macrophages, this feature is an important focus for further studies.

## Materials and Methods

Molecular weight markers for gel filtration and Superdex™-200 10/30HR column were purchased from GE Healthcare, USA. SDS, GdmCl, urea, trypsin, acrylamide, Bis-acrylamide and TEMED were procured from Sigma Chemical, New Delhi, India. Molecular weight markers for SDS-PAGE were purchased from MBI-Fermentas, Hanover, Maryland, USA. DTNB and Acetyl CoA were bought from SRL, Mumbai, India.

### Over-expression and purification of Eis protein

The *eis* (Rv2416c) gene was amplified, cloned, over-expressed and purified using Ni-NTA chromatography as described previously [13].

### Size exclusion chromatography

Gel filtration experiments were carried out on Superdex™-200 10/30HR (Manufacturer's exclusion limit 600 kDa for proteins) on AKTA FPLC. The column was equilibrated and run with 50 mM Tris Cl (pH 8.0), 100 mM NaCl, 2.0 mM EDTA, 2.0 mM βME buffer. Standard molecular weight markers were also run under the same conditions. Aldolase (176 kDa), Catalase (219 kDa), Ferritin (416 kDa), Thyroglobulin (699 kDa) were used for molecular weight determination. Blue dextran (2000 kDa) was used for detecting void volume. 1.0 mg of purified Eis protein was loaded on the column and run at 26°C, with a flow rate of 0.1 ml/min and detection at 280 nm. In GdmCl denaturation experiments, 1.0 mg Eis protein was incubated over night at 4°C in the presence of different concentration of GdmCl before loading onto the column and run at 25°C, with a flow rate of 0.1 m/min and detection at 280 nm.

### SDS-Polyacrylamide Gel Electrophoresis (PAGE) assay

10 µg each of boiled and un-boiled samples of Eis, containing 1% SDS in 0.125 M Tris (pH 6.8), was analysed by SDS-PAGE in the SDS-PAGE assay [23] using 10% Acrylamide gels and 0.1% SDS in Tris/Glycine buffer (pH 8.0) as the running buffer. The gels were then stained using Coomassie Brilliant Blue R250.

### Circular Dichroism (CD) measurements

CD measurements were carried out on a Jasco J810 spectropolarimeter calibrated with Ammonium (+)-10–Camphorsulfonate and fitted with a thermostatically controlled cell holder having an accuracy of ±0.1°C. The results are expressed as the mean residual ellipticity [MRE], in degree cm^2^/dmol, which is defined as [MRE] = θx 100×M_r_ /c×d×N_A_ where, θ is the observed ellipticity in degrees, c = protein concentration in mg/ml, and d = path length in cm, M_r_ = Protein molecular weight and N_A_ = number of amino acids. The percentage of α-helical content was calculated from MRE value at 222 nm as determined by K2D program (www.embl-heidelberg.de/~andrade/k2d.html). The CD spectra were recorded at protein concentration of 5.0 µM with a 1 mm cell at 25°C for far and near UV measurements.

For SDS interaction studies, protein samples were incubated in the presence of increasing concentration of SDS at 25°C for overnight before recording the spectra. Three individual scans were averaged for each spectrum. The values were normalized by subtracting the baseline recorded for the phosphate buffer having the same concentration of SDS under similar conditions. The 20 mM phosphate buffer was used for recording CD spectra.

For effect of temperature on the stability of protein, Eis was incubated at 60°C, 65°C and 70°C±0.4°C (as determined by sample probe) and the CD of the sample was measured from 200 to 260 nm for 50 minutes.

### Guanidinium chloride, Urea and Thermal denaturation

For urea and GdmCl denaturation studies protein samples in 20 mM phosphate buffer were incubated in the presence of increasing concentration of GdmCl or Urea for 12 hours at 4°C before the measurements was made.

Thermal denaturation studies were performed by recording spectra of protein samples at various temperatures starting at 25°C up to 85°C, with a 5°C increment. Samples were incubated for 10 min at each temperature before recoding the spectra. Fraction of protein folded corresponding to fractional helicities observed at MRE values at 222 nm were calculated by the equation [24]: ([θ]^obs^ – [θ]^den^)/([θ]^nat^ – [θ]^den^), where [θ]^obs^ is the experimental observed mean residue ellipticity at 222 nm, [θ]^nat^ and [θ]^den^ are mean residue ellipticities at 222 nm when the protein is in native state (at 25°C, in phosphate buffer, pH 6.5) and in fully denatured state (at 85°C, in phosphate buffer, pH 6.5). Unfolding curves of CD were produced by plotting fraction of protein folded vs temperature. Thermal scan in presence or absence of SDS was taken at 222 nm at the speed of 1°C/min in the wavelength range of 250-195 nm. Each thermal denaturation experiment was repeated at least twice with fresh samples. In all the cases, after the heating experiment, the samples were tested for their transparency.

### Fluorescence spectroscopy

1.0 µM of the Eis protein was heated from 25°C to 100°C for 10 min and the fluorescence spectra were recorded with Varience spectrofluorimeter using a 1.0 cm path length quartz cell. Protein samples of concentration 0.5 µM were incubated at RT (25°C) for 4.0 h in the presence of SDS before recording the spectra. For tryptophan fluorescence of Eis, excitation at 280 nm and the emission from 300–450 nm was recorded with 5 nm slit width. The values obtained were normalized by subtracting the baseline recorded for the buffer having the same concentration of SDS under similar conditions. Three individual scans were averaged for each spectrum. The 20 mM phosphate buffer (pH 8.0) was used for recording the spectra.

### Analysis of Eis-Acetyl CoA interaction by Fluorescence spectroscopy

Fluorescence emission spectra were measured for Eis protein on a Variance spectroflurimeter using a 1-cm quartz cuvette at 25°C. The emission spectra were recorded over a wavelength of 300–400 nm with an excitation wavelength of 280 nm. Eis protein was allowed to equilibrate for 2 min in phosphate buffer (pH 8.0) having different concentrations of Acetyl CoA ranging from 5.0 µM to 100 µM before recording the spectra. The binding of Acetyl CoA to Eis resulted in quenching of tryptophan fluorescence. The slit width of 5 nm for excitation and emission were used and each spectrum recorded was an average of three scans. Data analyzed according to Stern-Volmer relationship [25] which is represented by equation, i.e., F0/F = 1+Ksv[Q] where F0 and F are fluorescent intentsities in the absence and presence of Acetyl CoA respectively, Ksv is the Stern-Volmer constant and Q is the quencher (Acetyl CoA) concentration.

Effect of temperature on the binding of Acetyl CoA to Eis was performed by recording the fluorescence spectra by heating the 1.0 µM Eis protein at various temperatures ranging from 25°C to 100°C for 10 min and then Acetyl CoA at 20 µM was allowed to equilibrate for 2 mins in phosphate buffer (pH 8.0) before recording the spectra.

### Aminoglycoside acetyltransferase activity assays

Eis protein was treated with 2.0 mM SDS for 4.0 hours at room temperature (25°C) and subjected to SEC on Superdex™-200 10/30HR (Manufacturer's exclusion limit 600 kDa for proteins) on AKTA FPLC. Fractions having respective hexamers, trimers and monomers were collected and used for aminoglycoside acetyltransferase activity. Acetyltransferase activity was determined spectrophotometrically by measuring the increase in λ412 due to the formation of 5-thio-2-nitrobenzoate (TNB) resulting from the reaction between the sulfhydryl group of the product of the acetyltransfer reaction, CoA-SH, and 5,5′ dithiobis(2-nitrobenzoic acid) (DTNB) as described previously [26]. In short, 1.0 µg of hexamers, trimers and monomers from respective fractions were added to the reaction mixture containing 20 mM phosphate buffer (pH 8.0), 150 µM acetyl-CoA, 1.0 mM kanamycin, 20 µM DTNB and incubated at 20°C for 10 minutes. Apparent production of coloured TNB due to the enzymatic activity of Eis was assayed at 412 nm. Triplicates sets of reactions were carried out for hexamers, trimers and monomers.

The effect of heat on enzymatic activity of Eis was determined by incubating at temperatures ranging from 20°C to 80°C for 10 min, centrifuged at 12,000 g for 5 min and then assayed for activity. Triplicate sets of reactions at each temperature were recorded.

### Effect of temperature on the survival of *M. smegmatis* due to Eis

Eis gene of *M. tuberculosis* H37Rv cloned into pMV261 vector was transformed into *M. smegmatis* (*M. smegmatis*/*Eis*). *M. smegmatis* transformed with pMV261 vector alone was included in every experiment as a negative control. *M. smegmatis* harbouring either Eis or vector was grown up to log phase at 37°C (OD_600_ equals 0.8); cells of respective cultures were incubated further at 37°C, 50°C, 60°C and 70°C for 10 min by transferring aliquots to a set of flasks maintained at the respective temperatures in a water bath. The temperature equilibrium was reached within 1 min of transfer. Survival of cells was determined by plating appropriate dilution in triplicates on to 7H11 agar plates containing 50 µg/ml kanamycin. The colony forming units (cfu) were counted after incubation at 37°C for 3 days.

### Intracellular survival assay

The intracellular survival assay using THP1 cell line was performed as described by Wei et al [7]. In short, suspension cultures of 10^5^ cells/ml/well of THP-1 were grown in RPMI 1640 supplemented with 10% FCS at 5.0% CO_2_, 37°C for 24 hours into a 24 well tissue culture plate and treated with PMA (30 ng/ml) to transform THP-1 into adherent state. PMA was removed and cells were washed twice with RPMI 1640 medium.

Log phase cultures of *M. smegmatis* growing in 7H9 medium supplemented with 0.2% glycerol, either harboring vector alone or Eis gene of *M. tuberculosis* H37Rv, were used for infecting THP1 macrophages. *M. smegmatis* transformed with pMV261 vector alone was included in every experiment as a negative control. The single mycobacterium cells were obtained by repeated washing and passing the culture through an insulin syringe and checking under visible microscope. On the assumption that an optical density of 1.0 at 600 nm (OD_600_) equals 3×10^8^ cfu per ml, an inoculum of 10^6^ cells/ml were heated at 37°C, 50°C and 60°C for 10 minutes. The heated inoculum of *M. smegmatis* either harbouring *Eis* gene of *M. tuberculosis* or vector alone was used to give a multiplicity of infection (moi) of 1∶10. No cytotoxicity was observed at this moi. The infection was done for 3.0 h at 37°C, 5% CO_2_ using an antibiotic free medium. After 3.0 h, THP1 cells were washed twice with RPMI 1640 medium alone and grown in RPMI 1640 medium supplemented with 10% FBS + Gentamycin (20 µg/ml) to get rid of extra cellular bacteria. The Gentamycin was used for 48 hours so as to prevent the extra cellular growth of any bacteria that might be released by premature lysis of infected THP1 cells. The survival was assayed at 3 h, 24 h and 48 h post infection. At respective time points, cells were washed twice with RPMI 1640 medium alone and cells (in triplicate wells) were lysed in distilled sterile water containing 0.05% SDS. The infection was quantified in triplicates by plating serially diluted cell lysate on 7H11 agar plates containing kanamycin. The cfu were counted after incubation at 37°C for 3 days.
